# Uniportal versus multiportal video-assisted thoracoscopic anatomical resection for NSCLC: a meta-analysis

**DOI:** 10.1186/s13019-020-01280-2

**Published:** 2020-09-09

**Authors:** Yueren Yan, Qingyuan Huang, Han Han, Yang Zhang, Haiquan Chen

**Affiliations:** 1 Department of Thoracic Surgery and State Key Laboratory of Genetic Engineering, Fudan University Shanghai Cancer Center, 270 Dong-An Road, Shanghai, 200032 China; 2 Institute of Thoracic Oncology, Fudan University, Shanghai, 200032 China; 3 State Key Laboratory of Genetic Engineering, School of Life Sciences, Fudan University, Shanghai, 200433 China; 4 Department of Oncology, Shanghai Medical College, Fudan University, Shanghai, 200032 China

**Keywords:** None-small cell lung cancer (NSCLC), Uniportal video-assisted thoracoscopic surgery (U-VATS), Meta-analysis

## Abstract

**Background:**

Uniportal video-assisted thoracoscopic surgery (U-VATS) has recently emerged as an alternative procedure for non-small cell lung cancer (NSCLC); however, whether U-VATS has advantages over multiportal VATS (M-VATS) remains unknown.

**Methods:**

We performed a systematic review of two databases (Pubmed and Web of Science) to search comparative studies of U-VATS and M-VATS anatomical pulmonary resection for NSCLC. Operative time, blood loss, number of resected lymph nodes, drainage duration, length of postoperative stay, pain in postoperative day 1(POD1) and conversion rates were retrieved to estimate the comparison of outcomes. A subgroup analysis stratified by study type (propensity-matched analysis and randomized-controlled trial versus non-propensity matched analysis) was performed.

**Result:**

A total of 20 studies with 4142 patients were included in this meta-analysis. U-VATS was performed on 1869 patients, whereas the other 2173 patients underwent M-VATS. This meta-analysis showed that there was no significant difference in operative time (U-VATS: 146.48 ± 55.07 min versus M-VATS: 171.70 ± 79.40 min, *P* = 0.81), blood loss (74.49 ± 109.03 mL versus 95.48 ± 133.67 mL, *P* = 0.18), resected lymph nodes (17.28 ± 9.46 versus 18.31 ± 10.17, *P* = 0.62), conversion rate (6.18% versus 4.34%, *P* = 0.14), drainage duration (3.90 ± 2.94 days versus 4.44 ± 3.12 days, *p* = 0.09), length of postoperative stay (6.16 ± 4.40 days versus 6.45 ± 4.80 days, *P* = 0.22), and pain in POD1 (3.94 ± 1.68 versus 3.59 ± 2.76, *p* = 0.07). Subgroup analysis showed the value of PSM and RCT group consistency with overall value.

**Conclusion:**

This up-to-date meta-analysis shows that the perioperative outcomes of U-VATS and M-VATS anatomical pulmonary resection are equivalent. In addition, the differences in long-term outcomes of these two approaches are still unclear. Thoracic surgeons should pay more emphasize on providing high-quality and personalized surgical care for patients, to improve the survival ultimately.

## Background

Since the first pneumonectomy was performed for a patient with non-small cell lung cancer (NSCLC) in 1933, surgery is one of the main treatment methods for NSCLC [1]. The past decades have witnessed continuous evolution and progress of surgical techniques, such as the utilization of segmentectomy and the development of video-assisted thoracoscopic surgery (VATS). Compared with the traditional thoracotomy, VATS has significant advantages, such as lower mortality, reduced postoperative pain, and better quality of life, which have been widely recognized by prospective randomized controlled trials [2–4]. Conventionally, the traditional VATS, known as multiportal VATS (M-VATS), was performed through 3 or 4 small incisions in the thoracic wall. In recent years, uniportal VATS (U-VATS) has become a new technique in thoracic surgery. Uniportal minimally invasive surgery has developed rapidly since Dr. Rocco first reported in 2004, expanding from the minor thoracic procedures such as wedge resection to complex operations such as lobectomy, segmentectomy, and even bronchial or pulmonary angioplasty [5].

There have already been numerous articles on the feasibility of U-VATS approach in the treatment of lung neoplasm. Quite a few studies showed no difference between the approaches in the key intra- and postoperative outcome [6–10]. Although some of them have demonstrated several potential advantages of the uniportal VATS technique, such as less intraoperative blood loss, shorter hospital stay, and reduced postoperative pain [11–13], the results of these studies were highly heterogeneous. For instance, Lin et al. indicated that U-VATS significantly increased operation time compared to M-VATS approach [14], while Bourdages-Pageau et al. held the idea that operation time was significantly decreased in U-VATS group [15]. One study reported shorter average hospital stay with uniportal VATS [16], while another showed it was longer [17]. Comparative clinical outcomes of U-VATS versus M-VATS still remain uncertain.

Here in, we conducted a comprehensive meta-analysis of prospective and retrospective studies, to compare the clinical outcomes of U-VATS and M-VATS anatomical pulmonary resection (lobectomy or segmentectomy) for NSCLC.

## Materials and methods

### Study selection

A literature review was conducted by 2 independent investigators (Y.R. Yan and Q.Y. Huang) through PubMed and Web of Science online data sources (up to December 31st, 2019), using the following search terms:

((uniport*) OR (single port) OR (single-port) OR (single incision)) AND ((Lung Neoplasms [MeSH Major Topic]) OR (pulmonary neoplasms) OR (lung cancer) OR (none small cell lung cancer) or (NSCLC)) AND ((VATS [MeSH Major Topic]) OR (video-assisted thoracoscopic surgery) OR (thoracoscop*) or (video assist*))

Additionally, reference lists of the identified papers were scanned for relevant articles to obtain further studies.

Studies that comply with the following criteria were included in this meta-analysis: (1) An unmatched or propensity score matched comparison between U-VATS and M-VATS anatomical pulmonary resection (lobectomy or segmentectomy); (2) Included at least one of the following outcomes was reported: operative time, resected lymph nodes, drainage duration, blood loss, length of postoperative stay (LOS), and pain in postoperative day 1 (POD 1); (3) Focused on NSCLC; (4) Published full text article; (5) Written in English. For articles with overlaps in study population from the same institution, we only included the one with the largest sample size.

Ultimately, 20 studies were included for quantitative analysis.

### Data extraction and assessment of methodological quality

Two independent investigators (Y.R. Yan and Q.Y. Huang) extracted data from all included studies by Microsoft Office Excel 2010 (Microsoft, Redmond, WA). In the case of conflicts, disagreements were adjudicated by a third impartial reviewer (Y. Zhang) and resolved by combined agreement. Baseline variables retrieved included the following: study name, first author, location, publication year, study period, study design, surgical procedure, and tumor stage. The following results were retrieved as comparative outcomes: operation time, blood loss, number of resected lymph nodes, conversion rate, drainage duration, length of postoperative stay and pain in POD1. Two independent investigators (Y.R. Yan and Q.Y. Huang) assessed the methodological quality of the pertinent studies according to the Newcastle Ottawa Scale (NOS), a scale of 0 to 9. Studies scored 6 or more were included in this article.

### Data analysis

This meta-analysis retrieved and analyzed data according to the preferred reporting items for systemic reviews and meta-analysis (PRISMA) statement [18]. Meta-analysis was performed using R Studio Version 3.6.1 Meta packages (version 4.9–7). The effective values of continuous variables (operation time, blood loss, number of resected lymph nodes, drainage duration, length of postoperative stay, and pain in POD1) were estimated by standard mean differences or weighted mean difference (SMD or WMD) with 95% confidence intervals (CI), while those of categorical variables (conversion rate) were estimated by odds ratio (OR) with 95% confidence intervals. We performed a subgroup analysis stratified by study type (randomized controlled trials (RCTs) & propensity matched (PSM) studies versus non-propensity matched (non-PSM) studies) in operation time, blood loss, number of resected lymph nodes, drainage duration, and length of postoperative stay. Statistical heterogeneity was evaluated by Cochrane Chi-square test, with I2 values of 25, 50 and 75% representing low, moderate, and high heterogeneity. A random-effect model was used if I2 > 25%, otherwise, a fixed-effect model was adopted. Funnel plots were used to graphically assess publication bias. Meanwhile, Egger’s test and Begg’s test were used to quantify the publication bias. A statistical difference was taken as two-sided *P* value < 0.05.

## Results

### Study selection and risk of bias assessment

**Fig. 1:**
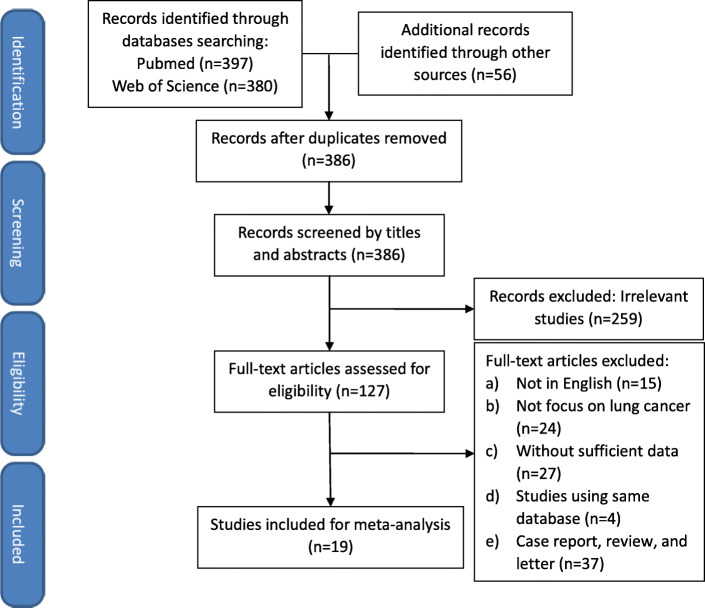
Flow chart detailing the search strategy and process of study selection

A total of 397 studies were identified from PubMed and 380 studies were searched from Web of Science online database by the previously mentioned electronic search strategy up to December 31, 2019. Upon a manual search and inspection of the reference lists of other systematic reviews and meta-analyses identified 56 additional relevant studies. After exclusion of duplicates, irrelevant studies or unoriginal studies, there were 127 studies remained and assessed for eligibility by screening the full text. Finally, 20 full-text studies reporting comparative clinical outcomes of U-VATS versus M-VATS met the inclusion criteria and were suitable for meta-analysis. The PRISMA flow chart describing the process of study selection is shown in Fig. 1.

The studies selected for this meta-analysis were conducted in 6 countries which were published before 31st December, 2019. Among these 20 studies, four of them were prospective studies; one of them was RCT; and seven of them were PSM studies. This analysis included 4142 patients, of which 1869 patients underwent U-VATS and 2173 patients underwent M-VATS. The quality of the included studies was assessed by the NOS scale and scores ranged from 6 to 8. Table 1 summarized basic characteristics and demographics of the included studies.

**Table 1:** Basic characteristics and demographics of the included studies

First Author	Country	Year of Publication	Study year	Retrospective/ Prospective	Study Type	Patients	Lobe	Seg	TNM^**8th**^ Stage	NOS score
Al-Ameri [17]	Sweden	2019	2016–2018	Retrospective	No	333	122/211	0/0	Stage I-IV	7
Bourdages-Pageau [15]	Canada	2019	2014–2017	Retrospective	PSM	722	247/247	0/0	T1N0M0	8
Chang [19]	China Taiwan	2016	2012–2014	Retrospective	No	121	26/55	3/2	T1-2N0M0	7
Chung [7]	South Korea	2015	2013–2014	Retrospective	No	150	90/60	0/0	T1-2N0M0	7
Dai [20]	China	2016	2013–2015	Retrospective	PSM	143	63/63	0/0	T1-3N2M0	7
French [21]	Canada	2016	2014–2015	Retrospective	PSM	100	40/42	10/8	T1N0M0	7
Han [22]	South Korea	2016	2006–2015	Retrospective	No	439	167/212	0/0/	Stage I or II	7
Heo [23]	South Korea	2017	2012–2015	Retrospective	PSM	104	32/32	0/0	T1-2N0-2M0	7
Hirai [24]	Japan	2019	2012–2019	Prospective	No	212	142/70	0/0	T1N0M0	7
Li [25]	China	2019	2015–2017	Retrospective	PSM	492	215/232	31/14	NG	8
Lin [14]	China	2016	2013–2014	Retrospective	No	67	21/46	0/0	NG	7
Liu [9]	China Taiwan	2016	2005–2014	Retrospective	No	442	100/342	49/47	NG	7
Liu [26]	China	2019	2015–2016	Prospective	No	328	166/162	0/0	T1N0M0	8
McElnay [8]	UK	2014	2012–2013	Retrospective	No	110	15/95	0/0	NG	7
Mu [16]	China	2015	2014–2015	Prospective	PSM	405	28/21	8/8	Stage I-III	8
Perna [27]	Spain	2016	2015–2016	Prospective	RCT	131	51/55	0/0	T1-2N0M0	8
Shen [28]	China	2016	2013–2014	Retrospective	PSM	396	100/100	0/0	T1-3N0M0	7
Song [29]	South Korea	2017	2011–2016	Retrospective	PSM	73	26/26	0/0	Stage I-III	7
Zhao [30]	China	2019	2013–2015	Retrospective	No	129	73/56	0/0	Stage I	7
Zhu [31]	China	2015	2014 Aug-2014 Oct	Retrospective	No	82	33/49	0/0	Stage I or II	7

*NOS score* Score of Newcastle Ottawa Scale, *RCT* randomized controlled trial, *PSM* propensity matched, *Lobe* Lobectomy, *NG* Not given, *Seg* Segmentectomy, *TNM*^*8th*^ 8th edition of TNM classification of lung cancer

### Operative outcomes

In this meta-analysis, the comparison of perioperative outcomes between U-VATS and M-VATS was estimated by intraoperative outcomes (operation time, blood loss, number of resected lymph nodes, and conversion rate) and postoperative outcomes (drainage duration, length of postoperative stay, and pain in POD1). Table 2 summarized the overall outcomes of uniportal and multiportal group.

**Table 2:** Summary of the perioperative outcomes between U-VATS and M-VATS in this meta-analysis

Comparative outcomes	Number of studies	Study group		SMD/WMD/OR	95%CI	P value	Heterogeneity (I^**2**^,P)	Meta-analysis model
		Uniportal	Multiportal					
**Intraoperative Outcomes**								
Operation time	18	1732	1967	-0.04	−0.33 ~ 0.26	0.81	I^2^ = 94%, p < 0.01	Random
Blood loss	14	1374	1590	−0.14	− 0.35 ~ 0.06	0.18	I^2^ = 86%, P < 0.01	Random
Number of resected lymph nodes	15	1391	1618	0.03	−0.08 ~ 0.13	0.62	I^2^ = 45%, *p* = 0.03	Random
Conversion rate	13	1375	1358	1.27	0.83 ~ 1.94	0.14	I^2^ = 13%, P = 0.32	Fixed
**Postoperative Outcomes**								
Drainage duration	18	1322	1411	−0.13	−0.27 ~ 0.02	0.09	I^2^ = 68%, p < 0.01	Random
Length of postoperative stay	12	1219	1271	−0.37	−0.81 ~ 0.08	0.22	I^2^ = 96%, p < 0.01	Random
Pain in POD1	5	234	313	−0.78	−1.61 ~ 0.05	0.07	I^2^ = 97%, p < 0.01	Random

### Operation time

**Fig. 2:**
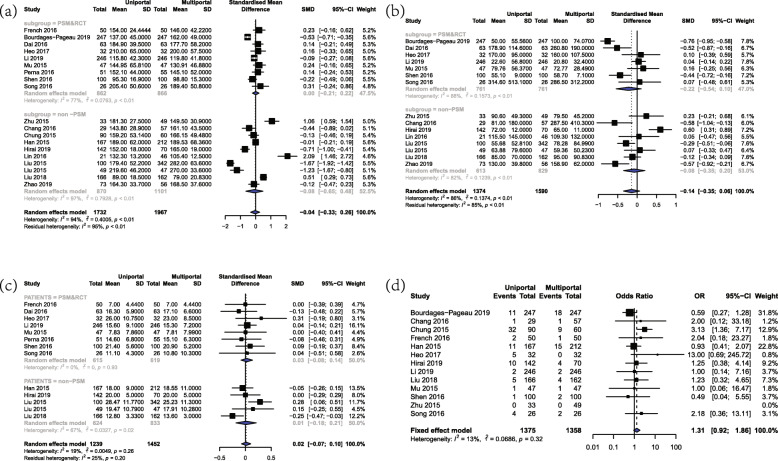
Forest plot of intraoperative outcomes for U-VATS and M-VATS groups. **a** Forest plot of operation time. **b** Forest plot of blood loss. **c** Forest plot of number of resected lymph nodes. **d** Forest plot of conversion rate

A total of 18 studies including 3699 patients provided comparative data on operative duration. The overall operation time was 146.48 ± 55.07 min and 171.70 ± 79.40 min in U-VATS and M-VATS group, respectively. The present meta-analysis revealed that there was no significant difference between U-VATS group and M-VATS group (SMD = -0.04, 95%CI = (− 0.33, 0.26), *P* = 0.81, Fig. 2a). Random-effect model was used due to the high heterogeneity (I^2^ = 94%, *P* < 0.01). Subgroup analysis of PSM&RCT studies further confirmed the comparable operation time between two approaches (SMD = 0, 95%CI = (− 0.21, 0.22)).

### Blood loss

Blood Loss was reported in 14 studies with a combination of 2964 patients. The overall blood loss was 74.49 ± 109.03 mL and 95.48 ± 133.67 mL in U-VATS and M-VATS group, respectively. The present meta-analysis indicated that there was no significant difference in blood loss between U-VATS group and M-VATS group (SMD = -0.14, 95%CI = (− 0.35, 0.06), *P* = 0.18, Fig. 2b). Random-effect model was used due to the high heterogeneity (I^2^ = 86%, *P* < 0.01). Subgroup analysis showed similar results in the PSM&RCT group (SMD = -0.22, 95%CI = (− 0.54, 0.10)).

### Number of resected lymph nodes

Totally, 15 studies including 3009 patients reported the comparative outcomes of number of resected lymph nodes, which were 17.28 ± 9.46 and 18.31 ± 10.17 in U-VATS and M-VATS groups, respectively. There was no significant difference in number of resected lymph nodes between U-VATS and M-VATS group (SMD = 0.03, 95%CI = (− 0.08, 0.13), *P* = 0.62, Fig. 2c). These results were further confirmed in the PSM&RCT subgroup (SMD = 0.03, 95%CI = (− 0.08, 0.14)). Random-effect model was used due to the moderate heterogeneity (I^2^ = 25%, *P* = 0.20).

### Conversion rate

In all, there were 13 studies including 2733 patients reporting conversion rate, which was defined as the rate of conversion to thoracotomy or need extra incisions. In U-VATS group, the total conversion rate was 6.18%, while the total value was 4.34% in M-VATS group. The meta-analysis result of conversion rate showed that there was no significant difference between U-VATS and M-VATS group (OR = 1.27, 95%CI = (0.83, 1.94), Fig. 2d). Fixed-effect model was used due to the low heterogeneity (I^2^ = 13%, *P* = 0.32).

### Drainage duration

**Fig. 3:**
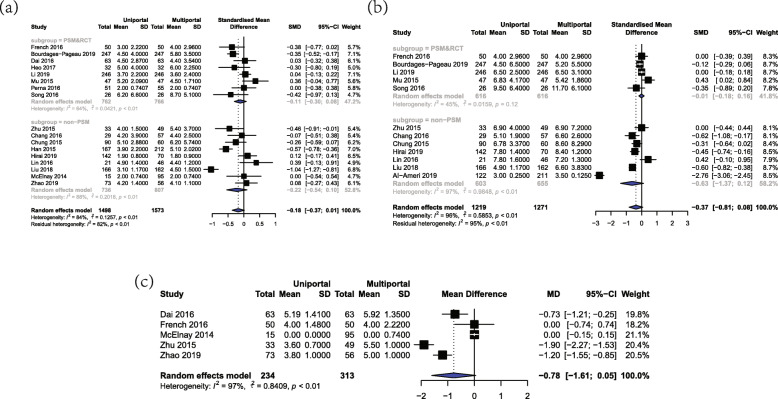
Forest plot of postoperative outcomes for U-VATS and M-VATS groups. **a** Forest plot of drainage duration. **b** Forest plot of length of postoperative stay. **c** Forest plot of pain in POD1

Drainage duration was defined as the period of time from the operation date to the extubation date. A total of 18 studies with a combination of 2743 patients provided comparative data on the length of drainage. The overall duration of drainage was 3.90 ± 2.94 days and 4.44 ± 3.12 days in U-VATS and M-VATS group, respectively. There was no significant difference between these two groups in drainage duration (SMD = -0.13, 95%CI = (− 0.27, 0.02), *P* = 0.09, Fig. 3a). These results were further confirmed in the PSM&RCT subgroup (SMD = -0.12, 95%CI = (− 0.30, 0.07)). Random-effect model was used due to the high heterogeneity (I^2^ = 68%, *P* < 0.01).

### Length of postoperative stay

There were totally 12 studies including 2490 patients reporting length of postoperative stay. The overall postoperative hospital stay was 5.67 ± 4.00 days in U-VATS group and 7.25 ± 5.10 days in M-VATS group. The present meta-analysis indicated that the length of postoperative stay has no significant difference between U-VATS and M-VATS group (SMD = -0.37, 95%CI = (− 0.81, 0.08), *P* = 0.22, Fig. 3b). According to subgroup analysis, length of postoperative stay in PSM&RCT group was (SMD = -0.01, 95%CI = (− 0.18, 0.16)). By the virtue of moderate heterogeneity (I^2^ = 64%, *P* < 0.01), random-effect model was applied to this analysis.

### Pain in POD1

A total of 5 studies including 547 patients provided comparative outcomes on pain scoring in postoperative day 1 (POD1). All these five included studies utilized the visual analogue scale (VAS) to evaluate pain in POD1, and the overall value of VAS was 3.94 ± 1.68 and 3.59 ± 2.76 in U-VATS and M-VATS group, respectively. Since all these studies utilized the same method to assess pain in POD1, the effective valuables of pain in POD 1 were estimated by WMD. The present meta-analysis indicated that the value of VAS has no significant difference between U-VATS and M-VATS group (WMD = -0.78, 95%CI = (− 1.61, 0.05), *P* = 0.07, Fig. 3c). Random-effect model was applied due to the high heterogeneity (I^2^ = 97%, *p* < 0.01).

### Publication bias

Funnel plots were utilized to graphically describe the publication bias of included studies in operation time, blood loss, number of resected lymph nodes, conversion rate, drainage duration, and length of postoperative stay. All funnel plots (See in Supplementary materials) showed a good symmetric distribution. Then Egger’s test and Begg’s test were used to quantize the publication bias, which demonstrated that there was no significant bias in each outcome.

## Discussion

This meta-analysis included 20 comparative studies reporting perioperative outcomes between U-VATS and M-VATS in 4142 patients with NSCLC. Compared with previous studies, this meta-analysis, which included the latest studies, has been the largest one on the comparative clinical outcomes between U-VATS and M-VATS anatomical resection for NSCLC so far. Our meta-analysis showed that there was no significant difference between U-VATS and M-VATS in operation time, blood loss, number of resected lymph nodes, conversion rate, drainage duration, length of postoperative stay and pain in POD1.

Recently, several meta-analyses had compared the perioperative outcomes of U-VATS and M-VATS [32–37]. Some of the previous meta-analyses elucidated better perioperative outcomes on U-VATS. For instance, Yang X.Y. et al. reported that patients in U-VATS group had a significant reduction with regard to blood loss, length of stay, and pain in POD1 compared with patients undergoing M-VATS approach [32]. Yang Z. et al. reported that U-VATS approach significantly reduced length of hospital stay against M-VATS approach [37]. By contrast, our meta-analysis demonstrated that there was no significant difference between U-VATS and M-VATS approach on these results, which showed that U-VATS approach has limited advantage over M-VATS in perioperative outcomes. The difference between this meta-analysis and the previous ones is mainly caused by following reasons. First of all, several previous meta-analyses were not focused on non-small cell lung cancer and included many studies on benign diseases. Compared with surgery for benign diseases, lung cancer surgery requires a radical resection of the primary lesion and lymph node dissection. Therefore, the scope of surgery and the degree of trauma are relatively large. In this situation, the perioperative outcomes of U-VATS may be better. In addition, the present meta-analysis is focused on anatomical pulmonary resection segmentectomy or lobectomy for patients with NSCLC, while some previous meta-analyses included patients undergoing wedge resection.

Both Yang Z. et al. and Yang X.Y. et al. showed that U-VATS achieved a significant reduction in the length of stay. This present analysis adopted length of postoperative stay as the parameter, and demonstrated no significant difference between U-VATS and M-VATS approach. Length of hospital stay includes length of postoperative stay and length of waiting for surgery. The latter depended on preoperative workup process, and could vary a lot according to protocols and criteria in different medical centers and treatment groups. Thus, the utility of length of postoperative stay may avoid potential biases and heterogeneity, and is much more objective reflecting the postoperative recovery.

There have been few studies reporting the long-term outcomes of U-VATS so far. Han et al. demonstrated that there was no significant difference between single-incision group, two-incision group, and three-incision group in both recurrence free survival and overall survival [22]. It is noteworthy that there was a study reporting a significant worse long-term survival in the U-VATS group compared with M-VATS group by Borro et al. in 2016. According to this study, Borro found that U-VATS led to a significant lower survival rate in tumor size (T2) and tumor stage (stage I) for patients with NSCLC by stratifying analysis. Besides that, Borro indicated that U-VATS approach was correlated with a higher risk (HR = 1.78) of death [38]. Due to the lack of studies with regard to long-term outcomes, we are unable to perform a meta-analysis of the long-term results. As surgical oncologists, the major impetus is always focused on optimal oncologic results [39]. A surgical procedure should never be performed by sacrificing the long-term survival. Although it is arbitrary to conclude that U-VATS result in poorer long-term outcomes based on only one study, thoracic surgeons should be cautious to avid uptake of this novel technique without well selecting the appropriate patients with lung cancer. Further studies of the survival of U-VATS are warranted.

VATS techniques are among the major progresses in the history of thoracic surgery beyond all doubt. U-VATS might have some potential advantages over M-VATS in reducing postoperative pain and drainage duration, even though these advantages are not significant. M-VATS, as a standard surgical procedure for NSCLC, has reliable safety and feasibility proved by several large-scale randomized controlled trials [2–4]. Comparatively, there is still lack of evidence that U-VATS can be less invasive without compromising the long-term survival. Innovation of surgical approach is of great importance, but minimizing the size and number of incisions is only one part of minimally invasive surgery (MIS). We believe that the utilization of MIS should lead to preserving normal organs, prolonging survival, and improving quality of life [40]. For instance, with the help of precise intraoperative frozen section diagnosis of pre-invasive lung adenocarcinoma, we are able to perform sublobar resection for these patients, to spare pulmonary function without impairing the survival [41].

There are some limitations in this meta-analysis. Firstly, U-VATS emerges as a novel surgical technique, so investigators have a propensity to publish positive outcomes to demonstrate the superiority or, at least, feasibility of U-VATS. Consequently, the equivalent results between two approaches reported in this meta-analysis should be quite conservative. Some of our included studies did not control the same surgeon to perform these two procedures, which would bring potential bias to the results of our study [7, 20, 21, 23, 26]. Our meta-analysis showed a high heterogeneity in the comparative outcomes (except conversion rates and number of resected lymph nodes). Several included studies mentioned the learning curve for U-VATS, and it might cause high heterogeneity. Besides, only four included studies are prospective in design, and the majority is retrospective which is of lower quality and inevitably introduce potential biases and heterogeneity to the results. We made a subgroup analysis between PSM&RCT studies and non-PSM studies, and found that the result of PSM studies was consistent with that of all included studies.

## Conclusions

To conclude, our results indicate that there is no significant difference in perioperative outcomes between U-VATS and M-VATS approaches in the treatment of NSCLC, which means that U-VATS, up to now, still cannot bring extra benefits over M-VATS on the perioperative recovery of patients. In addition, the differences in long-term outcomes of these two approaches are still unclear. Hence, U-VATS should be prudently chosen in the treatment of NSCLC.

## Supplementary information

**Additional file 1.**

## Data Availability

All data generated or analysed during this study are included in this published article.

## Abbreviations

NSCLC: Non-small cell lung cancer
U-VATS: Uniportal video-assisted thoracoscopic surgery
M-VATS: Multiportal video-assisted thoracoscopic surgery
POD1: Postoperative day 1
FEV1: Forced expiratory volume in one second
NOS: Newcastle Ottawa Scale
PRISMA: Preferred reporting items for systemic reviews and meta-analysis
PSM: Propensity-matched
RCT: Randomized controlled trial
SMD: Standard mean difference
WMD: Weighted mean difference
OR: Odds ratio
VAS: Visual analogue scale

## References

[1] Horn L, Johnson DH (2008). Evarts a. Graham and the first pneumonectomy for lung cancer. J Clin Oncol.

[2] Bendixen M, Jorgensen OD, Kronborg C, Andersen C, Licht PB (2016). Postoperative pain and quality of life after lobectomy via video-assisted thoracoscopic surgery or anterolateral thoracotomy for early stage lung cancer: a randomised controlled trial. Lancet Oncol.

[3] Long H, Tan Q, Luo Q, Wang Z, Jiang G, Situ D, Lin Y, Su X, Liu Q, Rong T (2018). Thoracoscopic surgery versus thoracotomy for lung Cancer: short-term outcomes of a randomized trial. Ann Thorac Surg.

[4] Scott WJ, Allen MS, Darling G, Meyers B, Decker PA, Putnam JB, McKenna RW, Landrenau RJ, Jones DR, Inculet RI (2010). Video-assisted thoracic surgery versus open lobectomy for lung cancer: a secondary analysis of data from the American College of Surgeons oncology group Z0030 randomized clinical trial. J Thorac Cardiovasc Surg.

[5] Rocco G, Martin-Ucar A, Passera E (2004). Uniportal VATS wedge pulmonary resections. Ann Thorac Surg.

[6] Wang BY, Tu CC, Liu CY, Shih CS, Liu CC (2013). Single-incision thoracoscopic lobectomy and segmentectomy with radical lymph node dissection. Ann Thorac Surg.

[7] Chung JH, Choi YS, Cho JH, Kim HK, Kim J, Zo JI, Shim YM (2015). Uniportal video-assisted thoracoscopic lobectomy: an alternative to conventional thoracoscopic lobectomy in lung cancer surgery?. Interact Cardiovasc Thorac Surg.

[8] McElnay PJ, Molyneux M, Krishnadas R, Batchelor TJ, West D, Casali G (2015). Pain and recovery are comparable after either uniportal or multiport video-assisted thoracoscopic lobectomy: an observation study. Eur J Cardio-Thorac Surg.

[9] Liu CC, Shih CS, Pennarun N, Cheng CT (2016). Transition from a multiport technique to a single-port technique for lung cancer surgery: is lymph node dissection inferior using the single-port technique?Dagger. Eur J Cardio-thorac Surg.

[10] Mu JW, Gao SG, Xue Q, Zhao J, Li N, Yang K, Su K, Yuan ZY, He J (2015). A matched comparison study of Uniportal versus Triportal thoracoscopic lobectomy and sublobectomy for early-stage nonsmall cell lung Cancer. Chin Med J.

[11] Rocco G, Martucci N, La Manna C, Jones DR, De Luca G, La Rocca A, Cuomo A, Accardo R (2013). Ten-year experience on 644 patients undergoing single-port (uniportal) video-assisted thoracoscopic surgery. Ann Thorac Surg.

[12] Ng CS, Kim HK, Wong RH, Yim AP, Mok TS, Choi YH (2016). Single-port video-assisted thoracoscopic major lung resections: experience with 150 consecutive cases. Thorac Cardiovasc Surg.

[13] Feng M, Shen Y, Wang H, Tan L, Mao X, Liu Y, Wang Q (2014). Uniportal video assisted thoracoscopic lobectomy: primary experience from an eastern center. J Thorac Dis.

[14] Lin F, Zhang C, Zhang Q, Cheng K, Zhao Y (2016). Uniportal video-assisted thoracoscopic lobectomy: an alternative surgical method for pulmonary carcinoma. Pak J Med Sci.

[15] Bourdages-Pageau E, Vieira A, Lacasse Y, Figueroa PU. Outcomes of Uniportal vs Multiportal Video-Assisted Thoracoscopic Lobectomy. Semin Thorac Cardiovasc Surg. 2020;32(1):145–51. 10.1053/j.semtcvs.2019.05.021 31150825

[16] Mu JW, Gao SG, Xue Q, Mao YS, Wang DL, Zhao J, Gao YS, Huang JF, He J (2016). A propensity matched comparison of effects between video assisted thoracoscopic single-port, two-port and three-port pulmonary resection on lung cancer. J Thorac Dis.

[17] Al-Ameri M, Sachs E, Sartipy U, Jackson V (2019). Uniportal versus multiportal video-assisted thoracic surgery for lung cancer. J Thorac Dis.

[18] Moher D, Liberati A, Tetzlaff J, Altman DG (2009). Preferred reporting items for systematic reviews and meta-analyses: the PRISMA statement. PLoS Med.

[19] Chang JM, Kam KH, Yen YT, Huang WL, Chen W, Tseng YL, Wu MH, Lai WW, Gonzalez-Rivas D (2016). From biportal to uniportal video-assisted thoracoscopic anatomical lung resection: a single-institute experience. Medicine.

[20] Dai F, Meng S, Mei L, Guan C, Ma Z (2016). Single-port video-assisted thoracic surgery in the treatment of non-small cell lung cancer: a propensity-matched comparative analysis. J Thorac Dis.

[21] French DG, Thompson C, Gilbert S (2016). Transition from multiple port to single port video-assisted thoracoscopic anatomic pulmonary resection: early experience and comparison of perioperative outcomes. Ann Cardiothorac Surg.

[22] Han KN, Kim HK, Choi YH (2017). Midterm outcomes of single port thoracoscopic surgery for major pulmonary resection. PLoS One.

[23] Heo W, Kang DK, Min HK, Jun HJ, Hwang YH (2017). Feasibility and safety of single-port video-assisted thoracic surgery for primary lung Cancer. Korean J Thorac Cardiovasc Surg.

[24] Hirai K, Usuda J (2019). Uniportal video-assisted thoracic surgery reduced the occurrence of post-thoracotomy pain syndrome after lobectomy for lung cancer. J Thorac Dis.

[25] Li J (2019). Uniportal video-assisted thoracic surgery could reduce postoperative thorax drainage for lung cancer patients. BMC Anesthesiol.

[26] Liu Z, Yang R, Shao F (2019). Comparison of postoperative pain and recovery between single-port and two-port thoracoscopic lobectomy for lung Cancer. Thorac Cardiovasc Surg.

[27] Perna V, Carvajal AF, Torrecilla JA, Gigirey O (2016). Uniportal video-assisted thoracoscopic lobectomy versus other video-assisted thoracoscopic lobectomy techniques: a randomized study. Eur J Cardio-thorac Surg.

[28] Shen Y, Wang H, Feng M, Xi Y, Tan L, Wang Q (2016). Single- versus multiple-port thoracoscopic lobectomy for lung cancer: a propensity-matched studydagger. Eur J Cardio-thorac Surg.

[29] Song KS, Park CK, Kim JB (2017). Efficacy of single-port video-assisted thoracoscopic surgery lobectomy compared with triple-port VATS by propensity score matching. Korean J Thorac Cardiovasc Surg.

[30] Zhao R, Shi Z, Cheng S (2019). Uniport video assisted thoracoscopic surgery (U-VATS) exhibits increased feasibility, non-inferior tolerance, and equal efficiency compared with multiport VATS and open thoracotomy in the elderly non-small cell lung cancer patients at early stage. Medicine.

[31] Zhu Y, Liang M, Wu W, Zheng J, Zheng W, Guo Z, Zheng B, Xu G, Chen C (2015). Preliminary results of single-port versus triple-port complete thoracoscopic lobectomy for non-small cell lung cancer. Ann Transl Med.

[32] Yang X, Li M, Yang X, Zhao M, Huang Y, Dai X, Jiang T, Feng M, Zhan C, Wang Q (2018). Uniport versus multiport video-assisted thoracoscopic surgery in the perioperative treatment of patients with T1-3N0M0 non-small cell lung cancer: a systematic review and meta-analysis. J Thorac Dis.

[33] Abouarab AA, Rahouma M, Kamel M, Ghaly G, Mohamed A (2018). Single versus multi-incisional video-assisted thoracic surgery: a systematic review and Meta-analysis. J Laparoendosc Adv Surg Tech Part A.

[34] Harris CG, James RS, Tian DH, Yan TD, Doyle MP, Gonzalez-Rivas D, Cao C (2016). Systematic review and meta-analysis of uniportal versus multiportal video-assisted thoracoscopic lobectomy for lung cancer. Ann Cardiothorac Surg.

[35] Lazar JF, Spier LN, Hartman AR, Lazzaro RS (2017). Standardizing Robotic Lobectomy: Feasibility and Safety in 128 Consecutive Lobectomies Within a Single Healthcare System. Innovations (Philadelphia).

[36] Ng CSH, MacDonald JK, Gilbert S, Khan AZ, Kim YT, Louie BE, Blair Marshall M, Santos RS, Scarci M, Shargal Y (2019). Optimal Approach to Lobectomy for Non-Small Cell Lung Cancer: Systemic Review and Meta-Analysis. Innovations (Philadelphia).

[37] Yang Z, Shen Z, Zhou Q, Huang Y (2018). Single-incision versus multiport video-assisted thoracoscopic surgery in the treatment of lung cancer: a systematic review and meta-analysis. Acta Chir Belg.

[38] Borro JM, Regueiro F, Pertega S, Constenla M, Pita S (2017). Comparative study of survival following Videothoracoscopic lobectomy procedures for lung Cancer: single- versus multiple-port approaches. Arch Bronconeumol.

[39] Taioli E, Lee DS, Lesser M, Flores R (2013). Long-term survival in video-assisted thoracoscopic lobectomy vs open lobectomy in lung-cancer patients: a meta-analysis. Eur J Cardio-thorac Surg.

[40] Cheng X, Onaitis MW, D'Amico TA, Chen H (2018). Minimally invasive thoracic surgery 3.0: lessons learned from the history of lung Cancer surgery. Ann Surg.

[41] Liu S, Wang R, Zhang Y, Li Y, Cheng C, Pan Y, Xiang J, Zhang Y, Chen H, Sun Y (2016). Precise diagnosis of intraoperative frozen section is an effective method to guide resection strategy for peripheral small-sized lung adenocarcinoma. J Clin Oncol.

