# Supplementary material for: Uniportal versus multiportal video-assisted thoracoscopic anatomical resection for NSCLC: a meta-analysis
Source: J Cardiothorac Surg. 2020 Sep 9;15:238. doi: 10.1186/s13019-020-01280-2 (PMC7488012; doi:10.1186/s13019-020-01280-2)
Supplement: Supplementary file 1 — Additional file 1. [file 13019_2020_1280_MOESM1_ESM.docx]

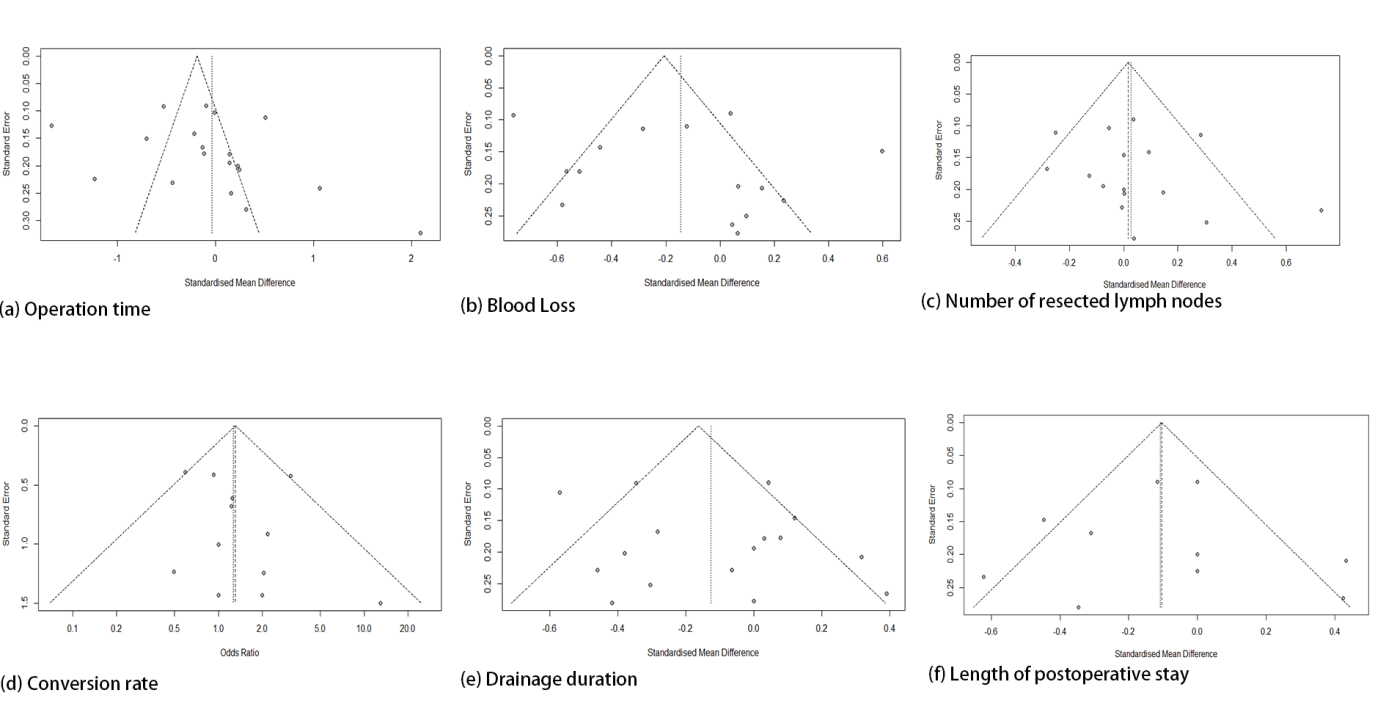


| **Comparative outcomes** | **Number of estimates** | **Begg's test (p)** | **Egger's test (p)** |
| --- | --- | --- | --- |
| Operation time | 18 | 0.09 | 0.23 |
| Blood loss | 14 | 0.59 | 0.37 |
| Number of resceted lymph nodes | 15 | 0.13 | 0.42 |
| Conversion rate | 13 | 0.22 | 0.44 |
| Drainage duration | 18 | 0.72 | 0.37 |
| Length of postoperative stay | 12 | 0.81 | 0.95 |
